# Deciphering moral intuition: How agents, deeds, and consequences influence moral judgment

**DOI:** 10.1371/journal.pone.0204631

**Published:** 2018-10-01

**Authors:** Veljko Dubljević, Sebastian Sattler, Eric Racine

**Affiliations:** 1 Department of Philosophy and Religious studies, North Carolina State University, Raleigh, North Carolina, United States of America; 2 Pragmatic Health Ethics Research Unit, Institut de recherches cliniques de Montréal, Montréal, Québec, Canada; 3 Institute for Sociology and Social Psychology, University of Cologne, Cologne, Germany; Universidad de Tarapaca, CHILE

## Abstract

Moral evaluations occur quickly following heuristic-like intuitive processes without effortful deliberation. There are several competing explanations for this. The ADC-model predicts that moral judgment consists in concurrent evaluations of three different intuitive components: the character of a person (Agent-component, A); their actions (Deed-component, D); and the consequences brought about in the situation (Consequences-component, C). Thereby, it explains the intuitive appeal of precepts from three dominant moral theories (virtue ethics, deontology, and consequentialism), and flexible yet stable nature of moral judgment. Insistence on single-component explanations has led to many centuries of debate as to which moral precepts and theories best describe (or should guide) moral evaluation. This study consists of two large-scale experiments and provides a first empirical investigation of predictions yielded by the ADC model. We use vignettes describing different moral situations in which all components of the model are varied simultaneously. Experiment 1 (within-subject design) shows that positive descriptions of the A-, D-, and C-components of moral intuition lead to more positive moral judgments in a situation with low-stakes. Also, interaction effects between the components were discovered. Experiment 2 further investigates these results in a between-subject design. We found that the effects of the A-, D-, and C-components vary in strength in a high-stakes situation. Moreover, sex, age, education, and social status had no effects. However, preferences for precepts in certain moral theories (PPIMT) partially moderated the effects of the A- and C-component. Future research on moral intuitions should consider the simultaneous three-component constitution of moral judgment.

## Introduction

People perform quick moral evaluations regularly, whether driving to work or just reading the news. For example, when faced with a person they consider evil (e.g., a Nazi), they might blame them. Individuals witnessing an action they think is wrong (e.g., rape), may be more likely intervene and seek subsequent punishment of the perpetrator. Facing a situation in which many individuals suddenly die, people might request that similar tragedies be prevented. And persons that fail to exhibit appropriate moral responses are viewed as having questionable integrity as moral agents. There is now mounting evidence suggesting that such moral evaluations occur quickly following heuristic-like processes without the engagement of effortful and deliberative cognitive systems [1,2]. But what are the mechanisms triggering these responses? The most plausible answer to this question is ‘moral intuitions’. Jonathan Haidt, a pioneer of research into intuitive moral psychology, defines moral intuition as “the sudden appearance in consciousness of a moral judgment, including an affective valence (good-bad, like-dislike), without any conscious awareness of having gone through steps of searching, weighing evidence, or inferring a conclusion” ([3], p. 818). But how does this moral judgment appear? And why are some moral judgments considered ‘intuitive’, while others, which are deduced from normatively and cognitively demanding moral theories, are ‘counterintuitive’ [4]? For example, when asked if lying is wrong, most people will confidently say that it is, yet when faced with a situation in which considerable harm might come from telling the truth (e.g., when lying to a serial killer who asks for the whereabouts of his intended victims), they might reluctantly conclude that lying is morally acceptable in that particular case. This flexibility of moral judgment has frustrated efforts at reaching a consensus on a single moral theory that would adequately explain and justify moral judgment, and at the same time has led to concerns that moral intuitions are unstable and sensitive to seemingly irrelevant influences (see [5]). Thus far, moral psychology and theory have provided several competing explanations about the nature of moral intuition and underlying cognitive mechanisms: Moral Foundations Theory [6], Universal Moral Grammar [7,8], and the Agent-Deed-Consequence (ADC) model of moral judgment [9–11]. Whereas tests of the two prior explanations have not yielded definitive support for the postulated hypotheses and while the evidence is far from being unequivocal or conclusive (see S1 and S2 Texts), we want to focus on the latter model, which has not been directly tested yet.

### Agent-Deed-Consequence model of moral judgment

The ADC-model [9–11] gives a fairly testable explanation for moral intuitions: it posits that moral judgment relies on positive and negative evaluations of three different components of moral intuitions: the character of a person (the Agent-component, A); their actions (the Deed-component, D); and the consequences brought about in a given situation (the Consequences-component, C). The theoretical approach underlying this model is grounded in the initial recognition that moral judgment involves multiple considerations that are difficult to compute. Moreover, most untrained individuals do not have explicit knowledge of philosophical ethics, and yet their intuitive moral judgments correspond to certain moral precepts implied in key ethical theories (e.g., see [12]). The ADC-model, following integrative approaches to moral theory [13,14] and empirical evidence noted above, considers the insights of three dominant ethical theories: virtue ethics, which focuses on the intentions and character of a person involved in a morally salient situation; deontology, which focuses on the analysis of certain actions that are prohibited or need to be undertaken as a duty; and consequentialism, which focuses on the balance of harms and gains resulting from the morally salient situation.

According to this integrative model, the moral evaluation of a situation can happen quickly and efficiently through the heuristic processing of morally salient cues. The heuristic processes substitute the overall moral judgment with more accessible information in distinct computations [9], and the outcomes of these are combined to form intuitive moral judgment. The ADC model suggests that this mostly happens unconsciously, but that conscious processes might monitor and correct judgments. The model predicts moral judgments to be positive if all three of the A-, D-, and C-components are positive and to be negative if all characteristics of the situation are evaluated as being negative. An important question, that will be explored in the following experiments, is what happens when the moral intuitions from the three components do not align. The model provides formulas for representing these situations, such as: [A+], [D-] and [C+] = [MJ+], where ‘+’ denotes ‘positive’, ‘-‘ denotes ‘negative’ and ‘MJ’ stands for moral judgment [9–11]. For instance, if the character and intentions of a person are good, and the action is good, individuals may be more likely to excuse bad consequences ([A+], [D+] & [C-] = [MJ+]). If the consequences are good and the action is done according to what duty demands, individuals may be more likely to forgive less than savory character traits and intentions ([A-], [D+] & [C+] = [MJ+]. Finally, the ADC-model explains how the three mentioned dominant moral theories rest on intuitive evaluations of the A-, D-, and C-components respectively. This implies that explicit moral precepts (i.e., concrete rules based on general principles) from these theories can be sufficiently dissociated from each other. The integrative approach also avoids “‘ethical blind spots’ and outlandish conclusions stemming from dogmatic application” of single-component theories ([9]:16), and provides a richer, multi-component, understanding of moral judgment.

#### Current study

In this study, which comprises two experiments, we provide a first empirical investigation of some of the predictions yielded by the ADC-model using a factorial survey describing different moral situations (vignettes) across experimentally varied components of the ADC-model. Previous empirical support for the model has been indirect in terms of post-hoc explanations of numerous empirical findings (e.g., [15]), which were not testing the predictions of the model. We undertook a significant amount of work to develop a novel experimental paradigm, to avoid falling prey to the pitfalls of “trolley-ology” [16]. Most dilemmas used in experimental and survey research on moral judgment consist of trolley problems (see [17]), which are a poorly suited tool for teasing details apart from conflicting ‘consequentialist and ‘non-consequentialist’ responses (see [4,18]). Furthermore, much of the literature overlooks the predominantly mundane character of moral judgment [19]–the research has gravitated quickly to striking and artificial cases. Instead, we generated more realistic situations which were tested and piloted extensively. In both experiments, we were particularly interested in simultaneously testing the following predictions of the ADC-model where the moral acceptability was expected to be higher if the description of the:

1. Agent (A) is positive instead of negative;
2. Deed (D) is positive instead of negative;
3. Consequence (C) is positive instead of negative.

Apart from immediate hypotheses stemming from the ADC-model, this study hoped to elucidate additional important research questions. To gain a deeper understanding of moral judgment and its underlying processes, we explored whether and how the effects of the three components may affect each other, i.e., their potential interaction effects. If different components result in congruent moral intuitions, e.g., if the good intentions of an agent are congruent with his/her good deed, a positive interaction effect might occur. However, moral theories also generate antagonistic predictions, and individuals might hold antagonistic moral precepts, which could reflect the fluid boundaries of different components of moral intuitions, e.g., if a bad deed results in positive consequences, conflicts of intuitions are possible (see [20,21]).

The ADC model assumes that the A-, D-, and C-evaluations are not only characteristic of moral judgment, but also stable discrete components of moral intuition. However, a body of ethics literature is concerned with the distinctiveness of components or even domains of moral intuition (i.e., are they genuinely different from each other?) and their stability (i.e., do they remain the same across individuals or over time?) [1,2]. Thus, we examined whether the effects of the three components found in experiment 1 are similar in experiment 2 that uses another design, as well as to what degree the results could be generalized to another situation.

Finally, if moral judgments are influenced by the characteristics of the situation, namely variations in A, D, and C, as well as the conscious or unconscious evaluations they might trigger, then both experiments should explore whether these are the *relevant* influences—or if other variables (e.g., characteristics of moral decision-makers such as their age, sex, education, socioeconomic status) have additional effects on moral judgment as predicted by influential theories [22]. In addition, moral decision-makers might have developed their own explicit or implicit moral preferences for how to behave, judge, or solve moral problems [21,23,24]. With a newly developed instrument, we want to explore whether and how these preferences shape the way individuals react to variations of A, D, and C in order to further our knowledge about moral judgment. This will be tested in experiment 2.

## Ethics statement

The ethics committees of the Institut de recherches cliniques de Montréal (approval number: 2015–13), and North Carolina State University (exempt status study number: 9362) approved this study.

## Pretests

We conducted factorial surveys with vignettes, which are an experimental means of investigating causal effects. These vignettes are short descriptions of morally significant situations. This approach combines ideas from classical experiments and survey methodology to counterbalance the weakness of each approach: it provides high internal validity due to their orthogonal design; allows the investigation of multiple factors; provides an active mode of measurement; avoids multi-collinearity; allows for causal explanations; and acts as a good substitute for manipulations in the real world [25,26].

In this project, we initially developed seven preliminary vignettes featuring moral dilemmas of differing ‘stakes’. Stakes here refers to either the mundane or the more severe nature of a given moral transgression. We chose to develop both high- and low-stakes moral dilemmas because the mundane character of moral judgment is easily overlooked, and the fact that both the ethics and empirical moral psychology literatures have tended to gravitate toward striking and artificial cases [19], thus leaving a large proportion of actual moral judgment more or less unexplored [27]. With the help of a series of pretests (i.e., an expert review, a cognitive pretest, and a quantitative pretest), we selected the most suitable vignettes and undertook iterative refinement to ensure and increase the validity and quality of the instruments and design.

### Expert review

We solicited written and oral feedback from experts (*N* = 4) with backgrounds in moral philosophy, applied ethics, survey methodology, and experimental moral psychology. We asked them for their thoughts on the aims of the project and the research design including the face validity of all measures, the plausibility of the vignettes, and the measurement concept. The feedback was assessed with structured questions (e.g., about the quality of each section of the study, with qualifications of the responses as well as suggestions). As a result of this process and through team discussions, we excluded one vignette due to its low plausibility. The experts indicated that the six remaining moral dilemmas were sufficiently plausible, had face validity in terms of measurement concept, and matched the overall goals of the study well. With the help of this feedback, several minor adjustments were made to the formulation of the instruments.

### Cognitive pretest

To further refine the instruments, we conducted cognitive pretest interviews (*N* = 18) with a convenience sample of nine females and nine males of different age groups (18–50) and educational levels. We used a think-aloud-technique and probing questions to evaluate the understanding and clarity of our vignettes, questions, and instructions. The interviews were conducted by a research assistant under the supervision of the lead author, and verbal informed consent was obtained before each session. The assistant collected answers to the survey and took notes during each interview. Following team discussions, two vignettes were excluded because respondents consistently asked for clarifications, suggesting that the vignettes were not sufficiently comprehensible. Four vignettes were deemed clear enough for further testing.

### Quantitative pretest

We tested the overall procedure and the scale properties with the help of a quantitative web-based pretest. Moreover, we wanted to test whether the experimental variations of the ADC-components in the vignettes are valid manipulations of the underlying concepts by testing their effect on moral acceptability. Furthermore, we wanted to test the convergent validity of our newly developed Preferences for Precepts Implied in Moral Theories (PPIMT) instrument which provides an operationalization of the preferences for specific precepts implied in the three key ethical theories listed above (namely, virtue ethics, deontology, and consequentialism) by examining their correlation with the self-identification of professional philosophers who endorse these theories. For this pretest, we invited members of several philosophy-related professional networks and newsletters (Philos-L, PHILOSOP, philosophy network on LinkedIn, and North Carolina Philosophical Society) to our study. Participation was completely anonymous and voluntary. Two hundred twenty-two people (*N*_Philos-L_ = 143; *N*_PHILOSOP_ = 47, *N*_LinkedIn_ = 13, and *N*_North Carolina-Philosophical Society_ = 19) visited the first survey-page and 209 (94.1%) consented to participate. Four participants were excluded because they either participated twice or did not respond to the question regarding prior participation. One-hundred-fifty-two (74.1%) of the participants, who consented and were not excluded, completed the survey. To maintain statistical power, we also use non-completers and those with missing values if they have valid values for the respective analysis.

Respondents received four vignettes with different moral problems (due to space limitations, we only show and discuss the results for the moral problems that were later used in the main study, see Table 1). We varied the information about the A, D, and C described in the dilemma in a 2x2x2x3-between-subjects design. Each of the factors A, D, and C had two levels (i.e., negative and positive valence), while the final factor indicates whether respondents had to evaluate the moral status of A, D, or C by stating whether each of the three components can be described as “bad”, “immoral”, “unethical”, “wrong”, and “horrible”. Response options for these five items ranged from 1 “not at all” to 10 “absolutely” (cf. [28–30]). To analyze the structure of these items, we used principal-component factor analyses with varimax rotation in each variation of the final factor. We found one-dimensional structures in each experimental arm (the Kaiser-Meyer-Olkin Measures ranged from 0.736 to 0.874, which indicated a good suitability of the data for structure detection; Cronbach’s *α*’s ranged from 0.90 to 0.98 (detailed results of the factor analyses and the reliability analyses are available upon request). For the items in each arm, we used regression factor scores for each factor (score 0 indicates an average moral evaluation of the A, D, or C, and 1 is the standard deviation). This has been done because some items are typically more important than others for explaining a certain construct. Thus, factor scores account for the different impact of each item—which would be not the case when using unweighted sum scores [31].

**Table 1:** Low- and high-stakes vignettes.

*Low-stakes (Syphilis)*
After stepping on a bloody needle, a man is examined by a doctor. During his medical examination, the doctor tells the man he suspects that the man has syphilis. This is a potentially life-threatening but curable blood-borne and sexually transmitted disease. The doctor takes a blood sample for further testing. During the past couple of years, the man has been **[A-: *cheating on* │A+: *loyal to*]** his wife. After returning from the doctor’s appointment, he decides to **[D-: *lie to her this time* │D+: *tell her the truth*]** about the doctor’s prognosis. Two weeks later, the doctor informs him that he is **[C-: *ill and his wife has the first symptoms of syphilis* │ C+: *healthy and it was a false alarm*]**.
*High-stakes (Airplane)*
During a flight from a small airport in southern United States, a jewel thief suddenly threatens the pilot of the 8-seat airplane with a gun. The jewel thief is wanted by the police. He orders the four other passengers not to move or speak, and informs them that he is hijacking the plane and diverting it to Mexico, in order to escape. He emphasizes that no one will be harmed if they comply. However, some of the passengers started murmuring, making the jewel thief increasingly nervous, leading him to point his gun from one person to another. A martial arts instructor is one of the passengers. He is driven by a strong desire to keep **[A-: *the stolen jewels* │ A+: *innocent people out of harm’s way*]**. If the jewel thief’s gun goes off, it could damage the sensitive instruments of the airplane, causing a fatal crash. The martial arts instructor frantically considers his options. He could either calm the jewel thief down or cripple him with a quick blow to the neck. In the end, the martial arts instructor decides to **[D-: *cripple* │ D+: *calm*]** the jewel thief. As a result, the four passengers and the pilot **[C-: *die* │ C+: *are saved*]**.

Text in square brackets indicates the three experimentally varied vignette dimensions with negative and positive valence of A, D, and C. In the survey, the text was neither bolded nor italicized.

The manipulation check analysis indicated that all negative valences of the treatments resulted in significantly higher numerical values regarding the judgment of A, D, and C (i.e., these components are judged as being more morally problematic, as in more “bad”, “immoral”, etc.) compared to the positive valences for one low-stakes vignette (*M*_A-_ = 0.72; *M*_A+_ = -0.41; *t*(47) = 4.37; *p*<0.001; *M*_D-_ = 0.73; *M*_D+_ = -0.70; *t*(51) = 7.25; *p*<0.001; *M*_C-_ = 0.38; *M*_C+_ = -0.27; *t*(57) = 2.18; *p* = 0.032, see S1 Table) and one high-stakes vignette (*M*_A-_ = 0.35; *M*_A+_ = -0.39; *t*(46) = 2.67; *p* = 0.011; *M*_D-_ = 0.45; *M*_D+_ = -0.71; *t*(52) = 4.91; *p*<0.001; *M*_C-_ = 0.55; *M*_C+_ = -0.47; *t*(59) = 4.50; *p*<0.001). Due to the failure of manipulation checks (results available upon request), we excluded the two other vignettes from the study.

*Preferences for Precepts Implied in Moral Theories (PPIMT)*: To assess respondents’ preferences for the precepts implied in the three dominant moral theories (i.e., virtue ethics, deontology, and consequentialism), we developed a new instrument with 15 items (five for each theory, see Table 2). These items were presented after asking the following question: “When thinking about what is moral or immoral in a situation, it is important to me whether the involved persons…” Response options ranged from 1 “disagree very much” to 7 “agree very much”. We used principal-component factor analysis with varimax rotation (see Sample A in Table 2).

**Table 2:** Factor loadings of the items of the Preferences for Precepts Implied in Moral Theories (PPIMT) based on principal component factor analysis with a varimax rotation (Eigenvalues >1) (*N*_*A*_ = 140 and *N*_*B*_ = 786).

Factor	1: PPIMT Virtue ethics		2: PPIMT Deontology		3: PPIMT Consequentialism	
Sample	A	B	A	B	A	B
have good or bad intentions	**0.833**	**0.811**	0.195	0.132	0.032	0.282
have good or bad goals	**0.837**	**0.805**	0.147	0.200	0.159	0.210
have good or bad aims	**0.866**	**0.860**	0.143	0.152	0.115	0.185
have good or bad motives	**0.900**	**0.867**	0.141	0.193	0.053	0.189
have good or bad interests	**0.638**	**0.783**	-0.051	0.261	0.190	0.225
respect or do not respect certain obligations	0.068	0.197	**0.851**	**0.793**	0.086	0.199
respect or do not respect certain rules	0.099	0.169	**0.776**	**0.814**	0.049	0.141
respect or do not respect certain responsibilities	0.212	0.216	**0.822**	**0.842**	-0.074	0.140
respect or do not respect certain duties	0.221	0.219	**0.862**	**0.870**	0.015	0.083
respect or do not respect certain norms	0.071	0.087	**0.788**	**0.854**	-0.080	0.105
make somebody end up worse or better off	0.014	0.267	0.026	0.123	**0.861**	**0.778**
cause happiness or suffering	0.140	0.255	-0.095	0.118	**0.861**	**0.805**
are helping or harming	0.177	0.327	0.054	0.159	**0.772**	**0.715**
cause benefits or costs	0.006	0.197	0.110	0.277	**0.886**	**0.615**
cause pleasure or pain	0.171	0.242	-0.090	0.190	**0.826**	**0.710**
*Proportion of explained variance*	23.7%	26.1%	23.3%	25.6%	24.3%	19.9%
*Cronbach´s α*	0.87	0.92	0.90	0.91	0.88	0.84

*N* = Number of observations. Kaiser-Meyer-Olkin Measure_A_ = 0.78; Kaiser-Meyer-Olkin Measure_B_ = 0.92.

We found a three-factor structure with five items that have substantial loadings on the theoretically implied factors and only low secondary loadings on other factors. The Kaiser-Meyer-Olkin Measure of 0.78 indicated a good suitability of the data for structure detection. Cronbach’s *α*’s indicate that the constructs can be measured reliably. For the analysis, we again used regression factor scores for each factor (score 0 indicates an average preference for precepts implied in the respective theory, and 1 is the standard deviation). Moreover, we tested the convergent validity of the scale. Therefore, we asked the members of several philosophy-related professional networks and newsletters how much they self-identify as virtue ethicists, deontologists, and consequentialists (on a scale from 1 “not at all” to 10 “absolutely”). We found that a person’s stronger self-identification with a certain theory was significantly correlated with their respective PPIMT underlying this theory (*r*_(134) *Self-identification as virtue ethicist*, *PPIMT-Virtue ethics*_ = 0.397, *p*<0.001); (*r*_(137) *Self-identification as deontologist*, *PPIMT-Deontology*_ = 0.477, *p*<0.001); and (*r*_(137) *Self-identification as consequentialist*, *PPIMT-Consequentialism*_ = 0.480, *p*<0.001), while all other correlations were statistically insignificant (see S2 Table), which indicates a high convergent validity of the PPIMT.

## Experiment 1

### Aims

As described above, the first experiment examined how variations in A, D, and C influence moral judgments in a low-stakes dilemma (featuring a case of possible syphilis contamination). Additionally, we explored the potential interaction effects of these variations and whether any differences in moral judgments occur across respondents of different age, sex, education, and socioeconomic status.

### Methods

#### Participants and study design

We invited participants via Amazon Mechanical Turk (AMT) to take part in a web-based study. AMT is a website that facilitates payment for completing surveys, and such samples have been shown to provide more reliable and representative data compared to student samples [32]. Participation was completely anonymous and voluntary. We offered .80 USD as monetary compensation after completion to motivate people to participate [33,34]. Six-hundred-seven people visited the first survey-page. Of these, 599 (98.7%) consented to participate, and of which 568 (94.8%) completed the survey. To ensure data quality, we excluded 21 persons who failed an attention check in which respondents are ostensibly asked about their favorite color [35]. Due to item non-response, the analytic sample comprises 525 respondents of which 46.5% are female (see Table 3 for all descriptive statistics).

**Table 3:** Descriptive statistics (*N*_*Experiment 1*_ = 525; *N*_*Experiment 2*_ = 786).

	*Mean*	*Standard deviation*	*Min*	*Max*
**Experiment 1**				
*Dependent variables*				
Moral judgment_SYPHILIS_	5.14	3.571	1	10
*Independent variables*				
Male (Ref. Female)	0.54	0.499	0	1
Age	35.36	10.771	18	75
Education (in years)	15.29	1.691	8	20
Subjective social status	4.45	1.612	1	8
**Experiment 2**				
*Dependent variables*				
Moral judgment_SYPHILIS_	5.05	3.507	1	10
Moral judgment_AIRPLANE_	7.05	2.826	1	10
*Independent variables*				
Male (Ref. Female)	0.56	0.497	0	1
Age	37.13	11.349	20	77
Education (in years)	15.41	1.683	12	20
Subjective social status	4.59	1.619	1	9
PPIMT_Virtue ethics_	0	1	-4.69	2.20
PPIMT_Deontology_	0	1	-3.40	1.98
PPIMT_Consequentialism_	0	1	-4.75	2.33

#### Instruments

*Experimental design*: In a 2x2x2 within-subject design, we varied the A, D, and C of the low-stakes vignette (see Table 1). Unlike previous work (e.g., [21]) which mostly contrasted actions and outcomes, or actions and omissions, our vignettes consistently provided positive and negative descriptions for A, D and C components. Every participant received all eight possible variations of this dilemma which allowed a test of the experimental treatments within each participant. The allocation of the eight vignettes was randomized for every participant.

*Moral judgment*: After each vignette, we asked the following question to assess respondent’s moral evaluation of the situation: “Taking all circumstances into consideration, [item 1: for you personally/item 2: for society], how morally acceptable is what the man did in this situation?” (for similar but distinct measures of moral acceptability, cf. [36–38]). Response options ranged from 1 “not at all” to 10 “absolutely”. Given the very high correlation of both items for the syphilis vignette (*r* = .941, *p*<0.001), we computed a mean score.

*Socio-demographic information*: We assessed gender (0 “female”; 1 “male”), age, education in years (degrees have been assigned to the average duration of this educational level), and subjective social status to capture one’s sense of their own place on the social ladder (measured by the MacArthur Scale of Subjective Social Status; 1 “lowest status”, 10 “highest status”; [39]).

#### Statistical analysis

Each respondent rated eight vignettes, which resulted in a hierarchical structure of the data. Ratings of respondents have correlated error terms that can result in underestimated standard errors. Multilevel models were used to allow us to test for the effects of the experimental treatments (vignette level) and socio-demographics (respondent level). We used Wald post-estimation tests to explore statistical differences between the three ADC-components. We used a procedure for probing the three-way interaction between A, D, and C, including the conditional effects of A, D, and C, as well as all two-way interactions [40]. Simple slopes were graphically reported along with tests for differences of each pair of simple slopes by applying a slope difference test [41]. We plotted the interactions in three different ways (by exchanging the variables on the *x*-Axis across the plots), which is useful for the interpretation of such higher order interactions ([40]:52). We use an α-level of *p*<0.05 for the test of statistical significance.

### Results of experiment 1

**Fig 1:**
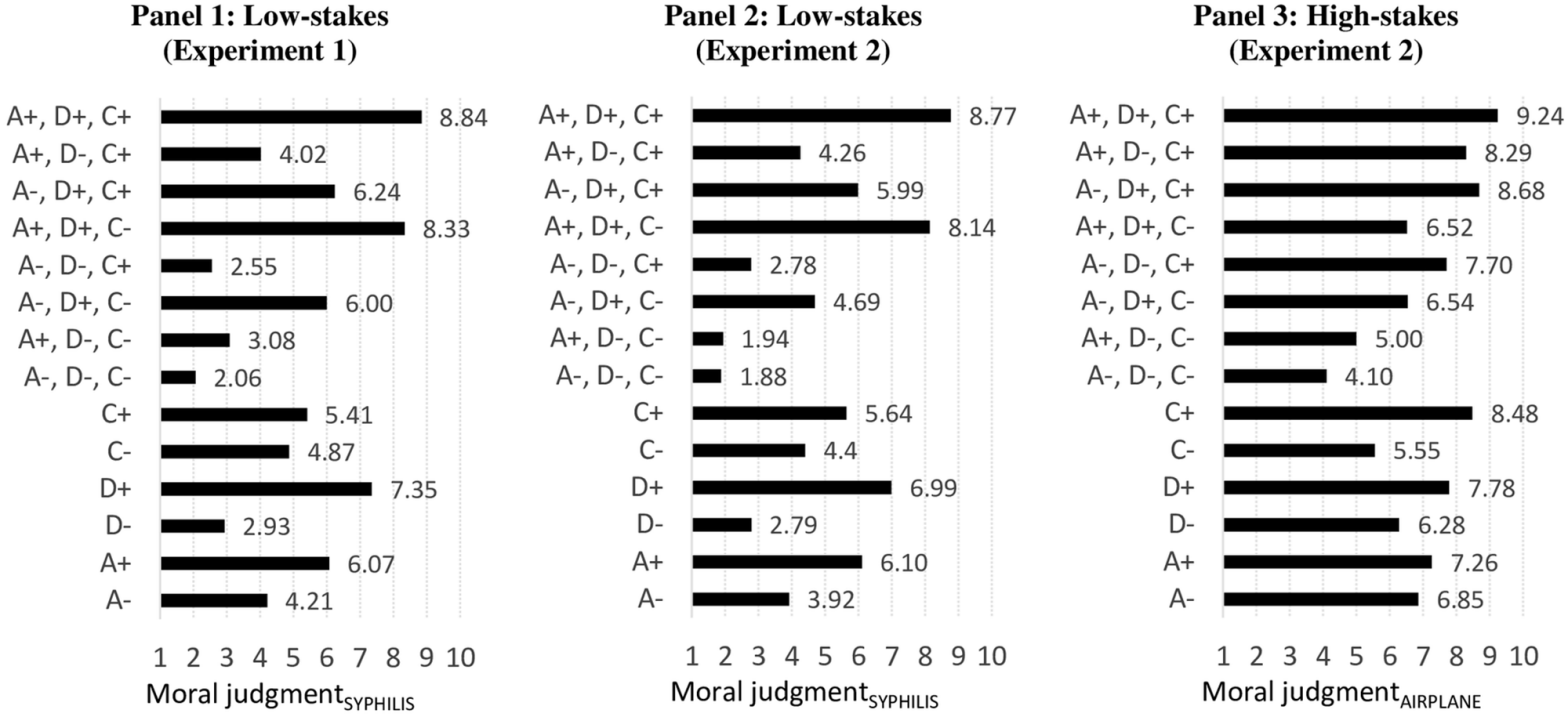
Mean values for moral judgments.

Panel 1 in Fig 1 shows the mean values of each treatment (lower part) and each treatment combination (upper part). An empty model without co-variates (not shown) shows that the vignette level accounts for 98.1% of the entire variance. Model 1 (M_1_) in Table 4 shows that if the man (i.e., the agent) is loyal to his wife (A+), respondents found the situation to be more morally acceptable compared to a situation where the agent has been described as an adulterer (A-)–as indicated by the significant positive *B*-coefficient (1.858, *p*<0.001, see also S3 Table which summarizes all findings of the ADC-components). When the man tells the truth about the doctor’s prognosis (D+) instead of lying (D-), the moral acceptability was higher (*p*<0.001). When it turned out that the man is healthy (C+) compared to when the man was ill and his wife has the first symptoms of syphilis (C-), the situation was judged as being more positive (*p*<0.001). Post-estimation Wald tests show that the effect of A was significantly smaller than the effects of D, but significantly larger than the effect of C, while the effect of D was significantly larger than the effect of C (all *p*<0.001). The measured respondent characteristics (i.e., sex (*p* = 0.541), age (*p* = 0.196), education (*p* = 0.170), and subjective social status (*p* = 0.877) had no significant effects and did not influence the effects of A, D, and C (model not shown, results available upon request).

**Table 4:** Multilevel models of the moral judgment regarding the low-stakes vignette on the experimental treatments and respondent characteristics (number of respondents = 525; number of vignettes = 4,200).

	M_1_	M_2_	M_3_
Agent (A+, Ref. A-)	1.858*** (24.90)	1.068*** (8.35)	1.023*** (6.99)
Deed (D+, Ref. D-)	4.442*** (59.27)	3.984*** (31.15)	3.939*** (26.68)
Consequence (C+, Ref. C-)	0.548*** (7.34)	0.537*** (4.20)	0.492*** (3.33)
A*D		1.219*** (8.25)	1.308*** (6.26)
A*C		0.361* (2.45)	0.450* (2.16)
D*C		-0.340* (-2.30)	-0.250 (-1.20)
A*D*C			-0.178 (-0.60)
Constant	1.742*** (19.78)	2.0434*** (18.89)	2.057*** (18.07)
**Variance components**			
Respondents—Level 2	1.069	1.084	1.085
(Standard error of the estimate)	(0.11)	(0.11)	(0.11)
Vignettes—Level 1	5.874	5.724	5.723
(Standard error of the estimate)	(0.14)	(0.13)	(0.13)
**Statistics**			
*AIC*	19821.3	19748.7	19750.4
*BIC*	19859.3	19805.8	19813.8
Bryk/Raudenbusch *R*^*2*^ (Level 2)	-3.523	-3.589	-3.589
Bryk/Raudenbusch *R*^*2*^ (Level 1)	0.533	0.543	0.543

* *p*<0.05,

** *p* < .01,

*** *p* < .001,

unstandardized coefficients with *t* statistics in parentheses. *BIC* = Bayesian information criterion; *AIC* = Akaike information criterion.

In M_2_, we tested all possible two-way interactions between treatments. We found positive interaction effects between A and D (*p*<0.001) and A and C (*p* = 0.014) as well as a negative interaction effect between D and C (*p* = 0.021). No three-way interaction between A, D, and C was found in M_3_ (*p* = 0.546). However, this model shows that the (conditional) interaction effect between D and C is no longer significant (*p* = 0.230) if A is negative.

**Fig 2:**
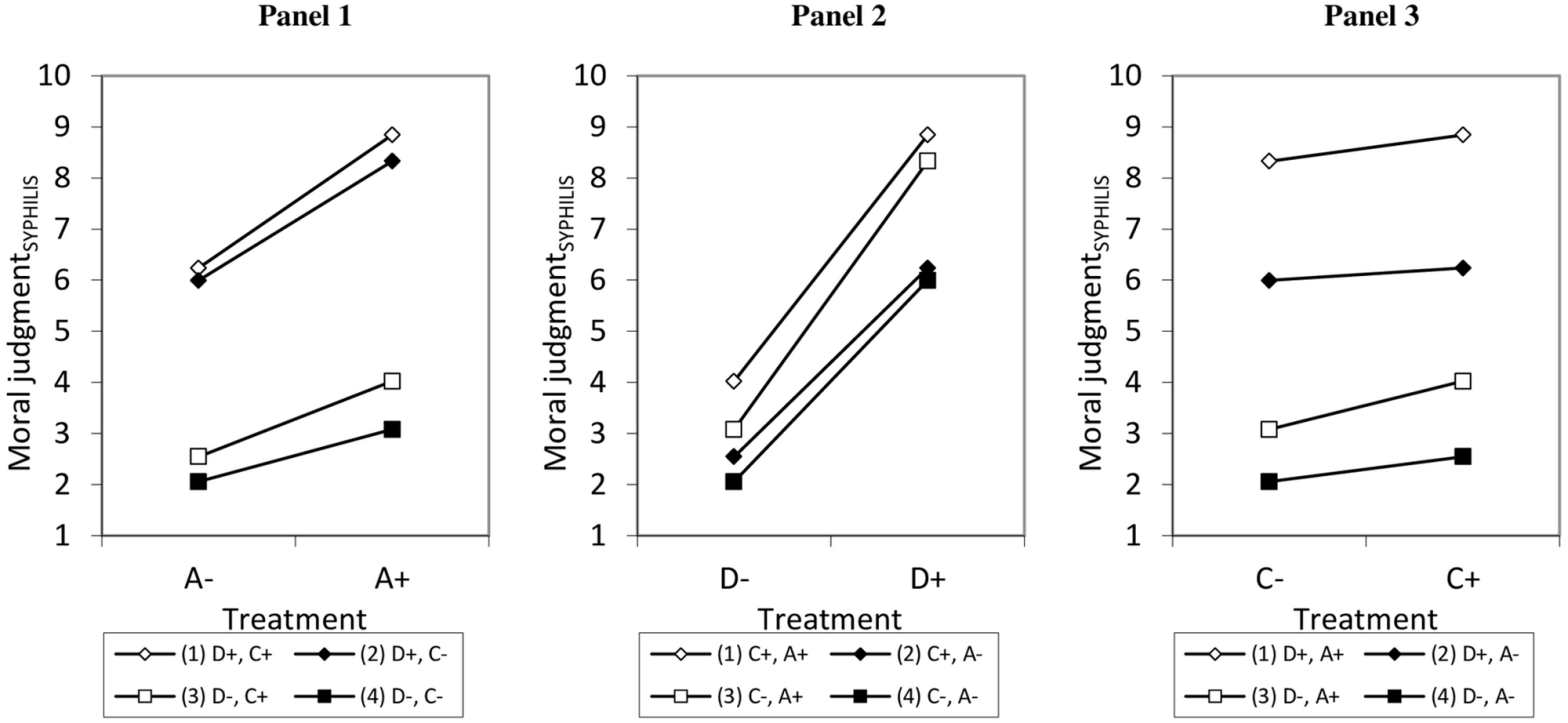
Predicted values for moral judgments regarding the low-stakes vignette (syphilis) depending on the experimental treatments and their interactions based on a linear regression model.

The S3 Table provides an overview of the complex interaction effects between A,D,C discovered in experiment 1 and which were further investigated in experiment 2. Fig 2 illustrates the effects of A, D, C, and their interactions and is accompanied by a simple slope analysis and slope difference tests to qualify and better understand the interaction effects. The simple slope analysis shows that A has significant positive effects in all treatment combinations (see lines 1–4 have positive ascents in Panel 1). Slope difference tests show that there is no interaction effect between A and C if D is positive (lines 1 and 2 are relatively parallel), but if D is negative, we find a positive interaction effect (line 3 is steeper than line 4). Thus, if D is negative, a positive C leads to a more positive effect of A (and vice versa). The positive interaction effect between A and D can be seen from the comparison of the following four lines: In situations with a positive C, the effect of A is larger if D is also positive rather than negative (line 1 is steeper than line 3), whereas in a situation with a negative C, the effect of A is also larger if D is positive rather than negative (line 2 is steeper than line 4). Moreover, when both D and C are positive, A has a larger effect than if D and C are negative (line 1 is steeper than line 4) and A has a stronger effect in a situation with a negative D and a positive C as compared to a positive D and negative C (line 2 is steeper than line 3), and that might be due to the stronger interaction effect between A and D compared to A and C.

Panel 2 shows that D has positive effects in all treatment combinations (see positive ascents of lines 1–4). It also shows the positive interaction effect between A and D from the perspective of D (line 1 is slightly steeper than line 2, and line 3 is steeper than line 4). Thus, a positive A leads to a more positive effect of D (and vice versa). When A is positive, D loses some strength if C is positive rather than negative (line 1 is flatter than line 3) indicating a conditional negative interaction effect between D and C, while when A is negative, D has similar effects irrespective of C (line 2 and 4 are relatively parallel). When both A and D are negative, D has a smaller effect than if A and C are both positive (line 4 is flatter than line 1). In a situation with a positive A and negative C, D has a stronger effect compared to one in which A is negative and C positive (line 3 is steeper than line 2).

Panel 3 shows that C mainly has positive effects (lines 1, 3, and 4 are ascending), C has no effect if D is positive and A is negative (line 2 is almost parallel to the x-axis). Furthermore, the negative interaction effect between D and C is significant if A is positive (in Panel 3 line 1 is slightly flatter than line 3), thus if A is positive, a positive D leads to a smaller positive effect of C (and vice versa). But there is no such interaction if A is negative (lines 2 and 4 are almost parallel). The effect of C also does not differ in situations with D and C being both positive compared to being both negative or compared to situations with a D positive and negative A (line 1 is relatively parallel to lines 4 and 2). If D is negative, C has a stronger effect in situations with a positive rather than negative A (line 3 is steeper than line 4), again indicating a conditional interaction effect between A and C. C also has a stronger effect if A is positive and D negative compared to if A is negative and D positive (line 2 is flatter than line 3).

### Discussion of experiment 1

The results of experiment 1 supported the most important prediction of the ADC-model: With a positive valence of A as compared to a negative valence of the A (being loyal vs. being an adulterer) the D (telling the truth vs. lying), and C (both the man and his wife wound up healthy vs. have syphilis), the situation was judged as being significantly more acceptable compared to when all three components had a negative valence. This is the first direct empirical corroboration of the ADC-model, and thus serves as ‘proof of principle’, which should be explored with more research to test predictions and additional hypotheses about the mechanisms underlying these different components of morality.

*Differences in component effect strength*: The strongest effect was observed in the variation of the D-component, followed by the A-component, whereas the C-component produced the smallest effects. This relative weakness of the C-component could be the result of, for example, a genuine difference in the intuitive strength of different components of moral judgment, or of the relative lack of moral impact for other people (i.e., the fact that it is a low-stakes scenario—it does not concern drastic outcomes, such as the death of innocent people).

*Interaction effects*: Our exploratory interaction analysis reveals a two-way interaction effect between A and D: If the agent was described as positive (i.e., loyal) rather than negative (i.e., an adulterer), the positive description of the action (i.e., telling the truth) had a stronger positive effect on moral judgment and vice versa. One interpretation of this effect might be that the description of the deed is congruent with the description of the agent (and vice versa). This supports a common belief that good people do good deeds (and that bad people do bad deeds). Qualitative data from the pre-test seem to indicate that this is the case at least with some responders. Namely, in the situation where the ‘loyal’ man decides to lie to his wife, several pretest participants verbalized their concerns along the following lines: ‘He might have been loyal because he didn’t have the chance to cheat; judging by the fact that he is lying, he ultimately would have cheated if able’. Therefore, a positive description of the agent confirms that the positive deed is not just a single instance of good behavior, but the agent’s overall stable disposition, which might lead to the stronger positive effect on moral judgment. Indeed, moral theories have long noted that congruence between intention and action is necessary for morality. For instance, Kant [42,43] famously argued that good deeds are not really moral if they are not motivated by good will.

We also found a conditional positive interaction effect between A and C: If A was positive rather than negative, the description of a positive C (i.e., that both the man and his wife are out of danger) had a stronger positive effect. But this interaction effect is conditional on the valence of D; it only occurs if D is negative, while no such effect exists if D is positive. This is in line with an opposing, but also common belief that good people sometimes do bad things to achieve significant good results.

Finally, we found a conditional negative interaction effect between D and C, i.e., if D was positive, the effect of a positive C was smaller (and vice versa), and this effect only occurred when A is positive but not if A is negative. However, when A was negative and D positive, we see that C has no effect. Generally speaking, deontology and consequentialism are moral theories which usually provide opposing ethical guidance, so some sort of reduction of effect is not surprising. The description of the agent could also be a ‘tie-breaker’ between C and D. For example, Dubljević & Racine [9] explain the difference between the trolley problem and the footbridge problem by taking into account implicit intuitions regarding the agent, which are not controlled (nor asked for) in most trolley dilemmas. In addition, intuitively deontological and consequentialist judgments are considered to be opposed in a large body of literature (see [4,21]). However, it has to be noted that the negative interaction effect between D and C is present only if A is positive—if A was negative, there was no two-way interaction between D and C, a finding for which we have no clear interpretation.

*Lack of socio-demographics effects*: We found no significant effects of the investigated socio-demographics (i.e., sex, age, education, and subjective social status) on moral judgment. It is important to note that the ADC-model only makes predictions regarding the relevance of A, D, and C. It makes no predictions regarding the effects of the individual characteristics of the moral decision-maker. However, some earlier models (e.g., [22]) have made such predictions. Our analysis, however, does not support this.

*Potential limitations*: This experiment used a within-subject design, and so every participant rated every variant of the situation. This could have produced learning effects, and contrast effects [44]. In addition, given that the vignette is low-stakes, the C-component might have had only a relatively small effect, which could change in a high-stakes situation. Experiment 2 aims to further explore the results of experiment 1 with another design, and test their generalizability to a more dramatic ethical problem.

## Experiment 2

### Aims

Experiment 2 was designed to address several potential limitations of experiment 1 and to explore the generalizability and robustness of its findings.

*Between-subject design*: In the previous within-subject experiment, every participant rated all eight variants of the low-stakes vignette. Exposing each participant to multiple vignettes might, for example, have elicited fatigue, learning, or contrast effects, and we minimized these effects in experiment 2 [25,26,44]. If the findings are similar in a between-subject design in which each participant reads only one vignette, this would rule out such effects.

*High-stakes situations*: Since it is assumed that the components of the model can play a role in different moral dilemmas, and our first experiment tested the model in a situation with relatively low-stakes, we wanted to test the ADC-model in a situation with higher stakes. Namely, based on the results of experiment 1, one might argue that the D-component is highly relevant in low-stakes scenarios, but that it could become irrelevant if the consequences were serious and harmful enough.

*Interaction effects*: We are also interested in whether the interaction effects that we found in our exploratory analysis are similar or different with this between-subject design across scenarios of varying levels of seriousness.

*Effects of PPIMT*: While situations have certain characteristics that are assumed to trigger moral judgment, certain characteristics of moral decision-makers such as their “ethical framework” [23] might also play a role in the perception of the situation and thus during moral judgment. This framework might include preferences for how to behave, judge, or solve moral problems. Such preferences can be seen as personality traits that are relatively stable over time and which are learned via socialization (see e.g., [45]). These preferences may embody the precepts implied by the three mentioned dominant moral theories: virtue ethics (e.g., “strive to be an honest person”), deontology (e.g., “obey rules, such as ‘never lie’”), and consequentialism (e.g., “maximize happiness and save lives with any means necessary”). Individuals might have preferences for different precepts of these theories which could serve as guidance for many morally salient situations in line with previous findings [21,24].

These preferences might work consciously or unconsciously: people with certain PPIMT might actively look for certain cues or significant symbols that help define the situation. Alternatively, they might be susceptible to implicit cues or symbols and thus, their preferences might create a certain accessibility, focus, or awareness for certain aspects of the situation, while other aspects might be less likely to be considered or might even be ignored. In this process, these preferences can help with processing information by structuring or framing the perception of characteristics of the situation (cf. [21,24]). We thus want to examine the effect of PPIMT on moral judgment, especially, the moderating role on the effects of the characteristics of the moral problem (i.e., the three ADC-components) on moral judgment. It can be argued that subscribing to such precepts or explicitly using them as tools moderates the effect of characteristics of the situation (cf. e.g., [21,23,24,46]). For example, stronger PPIMT_Virtue ethics_ might moderate the effect of the A-component (i.e., a virtuous agent might be judged more positively, while a non-virtuous agent might be judged more negatively). Moreover, someone with such strong preferences might tend to ignore variations of the D- or the C-components, or might pay less attention to them, thus giving D and C less weight in their overall moral judgment. Previous research explored a similar idea about how ‘ethical frameworks,’ in terms of a person’s predisposition towards either deontology or consequentialism, affects people’s moral judgment—and that it interacts with the parameters of the situation (see e.g., [46]) Another line of research has reported that a ‘consequentialist focus’ drives framing effects, whereas a ‘deontological focus’ decreases framing effects [21]. However, we do not know of any research that investigates the preferences for precepts implied in all three dominant moral theories at once.

### Methods

#### Participants and study design

Identical to experiment 1, we invited people via AMT to participate in our web survey under the same conditions. Of the 895 people who saw the first survey-page, 887 (99.1%) consented to participate, of which 874 (98.5%) completed the survey. We again excluded 19 people who failed the color attention check. Due to item non-response, the analytic sample comprised 786 respondents, of which 44.4% are female (see Table 3 for descriptive statistics).

#### Instruments

*Experimental design*: In experiment 2, we used a 2x2x2 between-subject design with the previous low-stakes vignette, and a high-stakes vignette (see Table 1) in which we again varied the A, D, and C of the dilemma. Every participant randomly received one variation of the low-stakes vignette and one variation of the high-stakes vignette of the eight possible variations for each.

*Moral judgment*: The moral judgment for both scenarios was assessed as in experiment 1. Due to the very high correlation of the “for you” and “for society”-items in each scenario (*r*_*SYPHILIS*_ = 0.93, *p*<0.001, *r*_*AIRPLANE*_ = 0.91, *p*<0.001), we again computed mean scores for this measure.

*Socio-demographic information*: Gender, age, education, and SES were assessed as in experiment 1.

*PPIMT*: We used the PPIMT instrument that we developed in the pretest (see above). We could replicate the three-factor structure and find similar Cronbach’s *α*’s and a slightly higher Meyer-Olkin Measure (see Sample B in Table 2). We again computed regression factor scores for each factor.

#### Statistical analysis

We used linear regression models to analyze the responses. Again, Wald post-estimation tests have been used to explore statistical differences between our three ADC-components. Simple slopes are plotted and tested against each other. We again use an α-level of *p*<0.05 to test for statistical significance.

### Results of experiment 2

Panel 2 in Fig 1 shows the mean values of each treatment in the low-stakes vignette (syphilis) and each treatment combination (respectively Panel 3 in the high-stakes vignette, i.e., airplane).

Similar to experiment 1, M_1_ in Table 5 shows that positive values of A, D, and C in the low-stakes scenario lead to a significantly more acceptable situation compared to respective negative values of A, D, and C (all *p*<0.001). In this scenario, post-estimation Wald tests again indicate that the effect of A was significantly smaller than the effect of D, but larger than the effect of C, whereas the effect of D was larger than the effect of C (all *p*<0.001).

**Table 5:** Linear regression models of the moral judgment regarding the low-stakes vignette (syphilis) and the high-stakes vignette (airplane) on the experimental treatments (number of respondents = 786).

	M_1_	M_2_	M_3_	M_4_	M_5_	M_6_
	Syphilis	Syphilis	Syphilis	Airplane	Airplane	Airplane
Agent (A+, Ref. A-)	2.050*** (11.28)	0.648* (2.02)	0.057 (0.15)	0.503** (3.07)	0.673* (2.36)	0.903** (2.74)
Deed (D+, Ref. D-)	4.170*** (22.91)	3.346*** (10.76)	2.810*** (7.83)	1.455*** (8.89)	2.210*** (7.71)	2.446*** (7.34)
Consequence (C+, Ref. C-)	1.212*** (6.67)	1.466*** (4.64)	0.904* (2.46)	2.927*** (17.87)	3.377*** (11.96)	3.602*** (11.05)
A*D		2.300*** (6.47)	3.388*** (6.62)		-0.472 (-1.45)	-0.931* (-2.00)
A*C		0.297 (0.84)	1.418** (2.73)		0.147 (0.45)	-0.311 (-0.67)
D*C		-0.677 (-1.90)	0.391 (0.77)		-1.022** (-3.14)	-1.467** (-3.20)
A*D*C			-2.083** (-2.94)			0.899 (1.38)
Constant	1.115*** (5.98)	1.606*** (6.67)	1.880*** (7.31)	4.560*** (27.54)	4.215*** (19.13)	4.097*** (17.33)
**Statistics**						
*AIC*	3702.3	3664.0	3657.3	3539.6	3533.8	3533.8
*BIC*	3720.9	3696.7	3694.7	3558.2	3566.4	3571.2
Adjusted *R*^2^	0.474	0.501	0.506	0.341	0.349	0.349

* *p*<0.05,

** *p* < .01,

*** *p* < .001.

Unstandardized coefficients with *t* statistics in parentheses. *BIC* = Bayesian information criterion; *AIC* = Akaike information criterion.

In M_2_, only a positive interaction effect can be found between A and D (*p*<0.001), while the interaction between A and C is not significant (*p* = 0.402). The interaction between C and D goes in the same direction as in experiment 1, but fails conventional levels of significance (*p* = 0.057).

**Fig 3:**
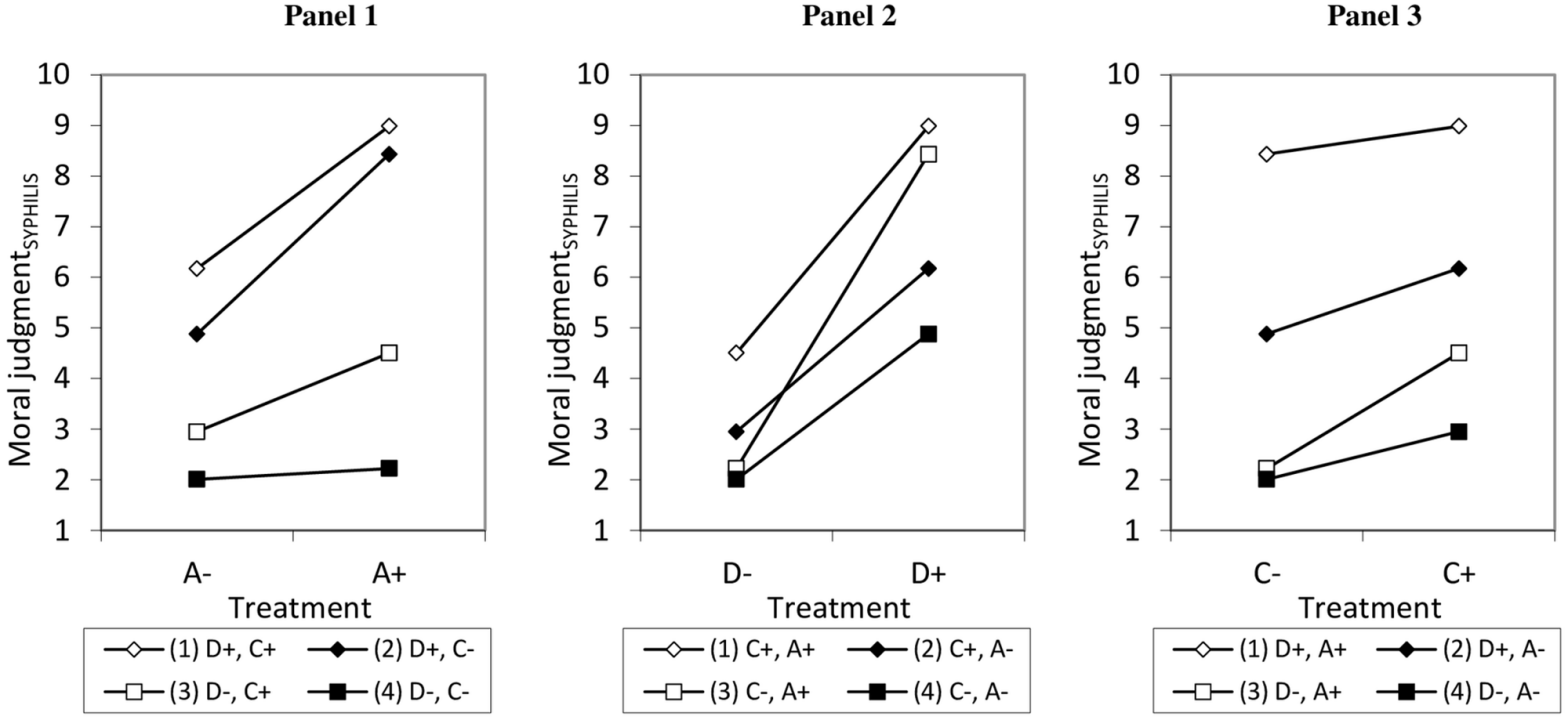
Predicted values for moral judgments regarding the low-stakes vignette (syphilis) depending on the experimental treatments based on linear regression models.

Complex interactions effects were further investigated following results of the first experiment. The moderate effect of the A-component was replicated in the low-stakes vignette but was weaker in the high-stakes vignette. As expected, the effect of the C-component was greater in the high-stakes vignette as opposed to the low-stakes vignette. M_3_ shows a significant negative three-way interaction between A, D, and C (*p* = 0.003). If C or D are positive, significant conditional interaction effects between A and D (*p*<0.001) and A and C (*p* = 0.006) occur, while there is no conditional interaction effect between D and C (*p* = 0.441). We again used simple slope analysis and slope difference tests to further examine the interaction effects (see Fig 3). The first simple slope analysis focuses on the conditional effects of A (see Panel 1). A has positive effects in three conditions (see lines 1–3). If D and C are negative, A has no effect (line 4 is almost parallel to the x-axis). As in experiment 1, there is no positive interaction effect between A and C if D is positive (in Panel 1, lines 1 and 2 are relatively parallel), but if D is negative, we again find a positive interaction effect (line 3 is steeper than line 4). Thus, if D is negative, a positive A leads to a more positive effect of C (and vice versa). As in experiment 1, a positive interaction effect between A and D can be seen: in situations with a positive C, the effect of A is larger if D is also positive rather than negative (line 1 is steeper than line 3), whereas in a situation with a negative C, the effect of A is also larger if D is positive rather than negative (line 2 is steeper than line 4). Moreover, when both D and C are positive, A has a larger effect than if D and C are negative (line 1 is steeper than line 4) and A has a stronger effect in a situation with a negative D and a positive C as compared to a positive D and negative C (line 2 is steeper than line 3), which might be due to the stronger interaction effect between A and D compared to A and C.

The component D has significant positive effects across all conditions (see ascending lines 1–4 in Panel 2). Additionally, there is a significant positive interaction effect between D and A if C is positive (line 1 is slightly steeper than line 2), it is also significant if C is negative (line 3 is steeper than line 4). Thus, as found in experiment 1, a positive A leads to a more positive effect of D (and vice versa). Similar to experiment 1, when A is positive, D loses some strength if C is positive rather than negative (line 1 is flatter than line 3) indicating a conditional negative interaction effect between D and C, while when A is negative, D has similar effects irrespective of C (line 2 and 4 are relatively parallel). When both A and D are negative, D has a smaller effect than if A and C are both positive (line 4 is flatter than line 1). In a situation with a positive A and negative C, D has a stronger effect compared to one in which A is negative and C positive (line 3 is steeper than line 2).

Panel 3 shows that C has positive effects in three treatment combinations (lines 2–4 have significant positive ascents). C, however, has hardly an effect when A and D are positive (line 1 fails conventional levels of significance). It was found that the negative interaction effect between D and C is significant if A is positive (line 1 is slightly flatter than line 3), but not if A is negative (lines 2 and 4 are relatively parallel). This means that we replicated the finding of experiment 1: if A is positive, a positive C leads to a smaller positive effect of D (and vice versa). As in experiment 1, if D is negative, C has a stronger effect in situations with a positive rather than negative A (line 3 is steeper than line 4), again indicating a conditional interaction effect between A and C, while no such interaction occurred if D is positive (lines 1 and 2 are relatively parallel). The effect of C also does not differ in situations with D and C being both positive compared to being both negative (line 1 is relatively parallel to lines 4). C again has a stronger effect if A is positive and D negative compared to if A is negative and D positive (line 2 is flatter than line 3).

M_4_ examines the main effects for the high-stakes vignette and finds significantly higher moral acceptability if: the martial art instructor is driven by the desire to keep innocent people out of harm’s way (A+) compared to the desire to keep the stolen jewels (A-) (*p* = 0.002); the martial arts instructor decides to calm the jewel thief (D+) instead of crippling him (D-) (*p*<0.001); and the four passengers and the pilot are saved (C+) vs. if they die (C-) (*p*<0.001). In this vignette, post-estimation Wald tests reveal that A has a significantly smaller effect than D and C, while the effect of D was smaller than the effect of C (all *p*<0.001).

**Fig 4:**
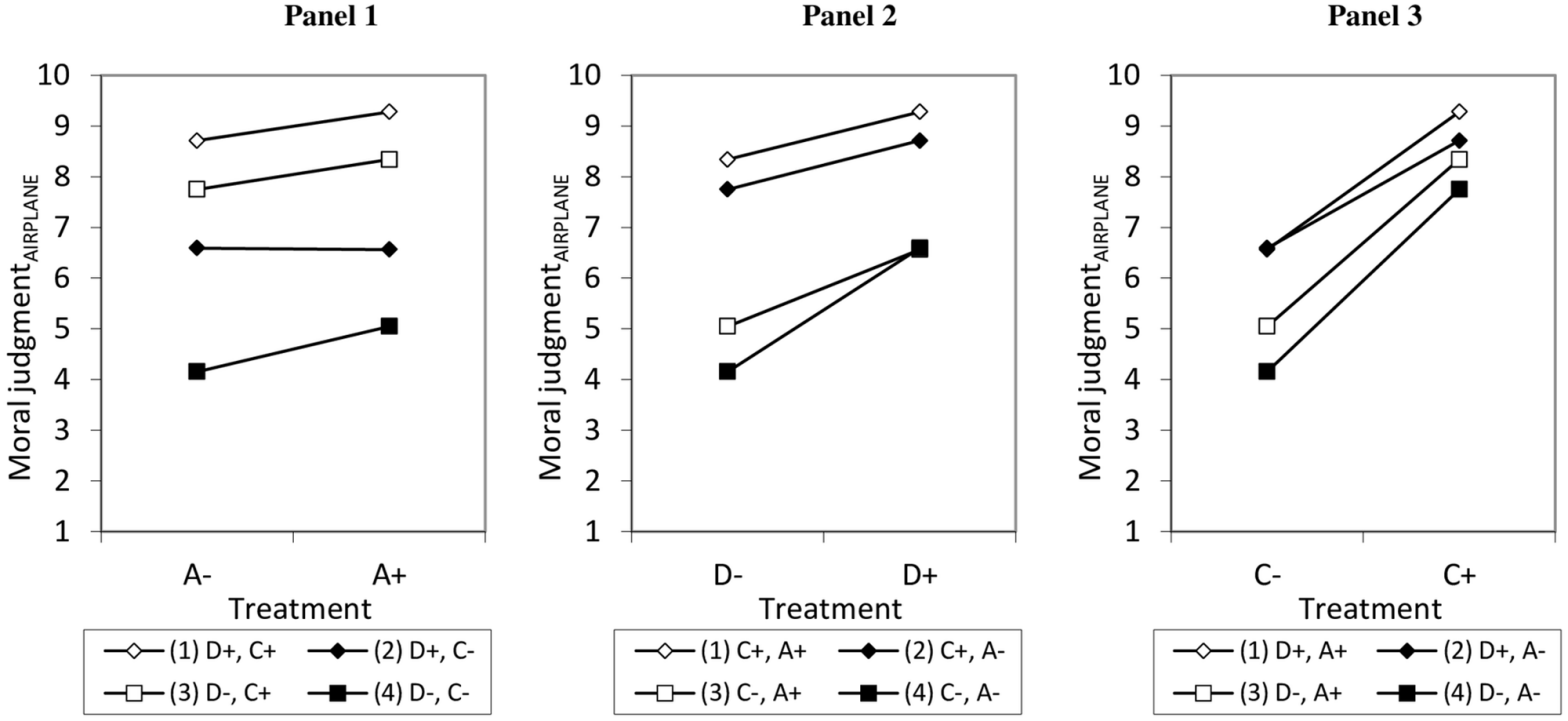
Predicted values for moral judgments regarding the high-stakes vignette (airplane) depending on the experimental treatments based on linear regression models.

M_5_ reveals that D has a smaller effect if C is positive and vice versa (*p*<0.002), while A and D (*p* = 0.148) and A and C (*p* = 0.651) do not interact significantly. M_6_ shows that the three-way interaction is insignificant (*p* = 0.168). It also shows a conditional negative interaction effect between A and D (*p* = 0.046) and D and C (*p* = 0.001). A further examination of the interaction effects with simple slope analysis shows that the effect of A is made less salient by the effects of D and C (lines 1–3 in Panel 1 of Fig 4 are relatively parallel to the x-axis). A only has a positive effect when D and C are negative (see positive ascent of line 4). The accompanying slope difference tests show that, unlike in the low-stakes vignette, no significant interaction exists between A and C—as can be seen from the relatively parallel lines 1 and 2, as well as 3 and 4. Also lines 1 and 3, 1 and 4, as well as 2 and 3 do not differ from each other, i.e., A has similar effects across these situations. The only exception is a stronger effect of A when D and C are negative as compared to when D is positive and C negative (line 4 is steeper than line 2)–indicating a conditional negative interaction effect between A and D.

Panel 2 shows that D has positive effects in all treatment combinations (lines 1–4 have positive ascents). Also unlike in the low-stakes vignette, the interaction effect between A and D is negative instead of positive, but only if C is negative (line 3 is flatter than line 4). Thus, if C is negative, D has a stronger effect if A is negative rather than positive. No significant interaction between A and D exists if C is positive (lines 1 and 2 are almost parallel). The conditional negative interaction effect between D and C implies from the perspective of D that D has a weaker effect when A is negative (line 2 is flatter than line 4), while no interaction exists when A is positive (lines 1 and 3 are almost parallel). Moreover, the effect of D is weaker if A and D are both positive as compared to both negative (lines 1 and 4 are almost parallel), while D has similar effects when A is negative and C positive compared to a positive A with a negative C (lines 2 and 3 are almost parallel).

Panel 3 shows that C has significant positive effects in all treatment combinations (see ascending lines 1–4). According to the slope difference test, no interaction exists between C and D if A is positive (the ascent of lines 1 and 3 is equal). If A is negative, C and D interact negatively (line 4 is steeper than line 2), thus, a positive D leads to a smaller positive effect of C (and vice versa)–while this was only the case for a positive A in the low-stakes vignette. No interaction exists between A and C (lines 1 and 2 as well as lines 3 and 4 are relatively parallel). C also has a similarly strong effect for situations when A and D are both positive or both negative (see relatively parallel lines 1 and 4). However, C has a stronger effect in the face of a positive A with a negative D than if A is negative and D positive (line 2 is flatter than line 3).

In M_1_ and M_3_ in Table 6, we test whether the moral judgments vary across personal characteristics.

**Table 6:** Linear regression models of the moral judgment regarding the low-stakes vignette (syphilis) and the high-stakes vignette (airplane) on the experimental treatments and respondent characteristics (N = 786).

	M_1_	M_2_	M_3_	M_4_
	Syphilis	Syphilis	Airplane	Airplane
Agent (A+, Ref. A-)	2.050*** (11.26)	2.013*** (11.02)	0.447** (2.77)	0.461** (2.92)
Deed (D+, Ref. D-)	4.161*** (22.82)	4.135*** (22.58)	1.369*** (8.46)	1.374*** (8.66)
Consequence (C+, Ref. C-)	1.170*** (6.40)	1.168*** (6.39)	2.986*** (18.23)	2.978*** (18.55)
Male (Ref. Female)	0.154 (0.83)	0.183 (0.98)	-0.208 (-1.26)	-0.150 (-0.93)
Age	0.004 (0.54)	0.004 (0.53)	0.004 (0.59)	0.006 (0.85)
Education (in years)	0.008 (0.13)	0.005 (0.09)	-0.023 (-0.56)	-0.032 (-0.62)
Subjective social status	0.055 (0.91)	0.064 (1.05)	0.047 (0.87)	0.052 (0.99)
PPIMT_Virtue ethics_	-0.167 (-1.83)	-0.402* (-2.28)	0.422*** (5.20)	0.179 (1.11)
PPIMT_Deontology_	-0.176 (-1.91)	0.013 (0.07)	-0.099 (-1.21)	-0.066 (-0.41)
PPIMT_Consequentialism_	0.005 (0.05)	-0.117 (-0.63)	-0.135 (-1.66)	-0.521*** (-3.31)
A*PPIMT_Virtue ethics_		0.438* (2.39)		0.216 (1.30)
D*PPIMT_Virtue ethics_		0.227 (1.24)		0.083 (0.50)
C*PPIMT_Virtue ethics_		-0.152 (-0.83)		0.117 (0.69)
A*PPIMT_Deontology_		-0.067 (-0.37)		-0.217 (-1.37)
D*PPIMT_Deontology_		-0.188 (-1.02)		-0.128 (-0.80)
C*PPIMT_Deontology_		-0.087 (-0.47)		0.341* (2.14)
A*PPIMT_Consequentialism_		-0.217 (-1.19)		-0.174 (-1.10)
D*PPIMT_Consequentialism_		0.353 (1.92)		0.123 (0.78)
C*PPIMT_Consequentialism_		0.035 (0.19)		0.958*** (5.85)
Constant	0.523 (0.58)	0.542 (0.60)	4.798*** (6.00)	4.643*** (5.94)
**Statistics**				
*AIC*	3706.7	3709.6	3519.3	3487.3
*BIC*	3758.1	3803.0	3570.6	3580.7
Adjusted *R*^2^	0.476	0.479	0.364	0.396

* *p*<0.05,

** *p* < .01,

*** *p* < .001.

Unstandardized coefficients with *t*-statistics in parentheses.

Neither the moral judgment in the low-stakes vignette nor in the high-stakes vignette is affected by sex (*p*_*SYPHILIS*_ = 0.407; *p*_*AIRPLANE*_ = 0.207), age (*p*_*SYPHILIS*_ = 0.593; *p*_*AIRPLANE*_ = 0.558), education (*p*_*SYPHILIS*_ = 0.894; *p*_*AIRPLANE*_ = 0.573), or subjective social status (*p*_*SYPHILIS*_ = 0.361; *p*_*AIRPLANE*_ = 0.387), nor is it affected by PPIMT_Virtue ethics_ (*p*_*SYPHILIS*_ = 0.068), PPIMT_deontology_ (*p*_*SYPHILIS*_ = 0.056; *p*_*AIRPLANE*_ = 0.229), and PPIMT_consequentialism_ (*p*_*SYPHILIS*_ = 0.958; *p*_*AIRPLANE*_ = 0.096). The only exception is that a stronger PPIMT_Virtue ethics_ leads to a higher moral acceptability in the high-stakes vignette (*p*_*AIRPLANE*_<0.001).

**Fig 5:**
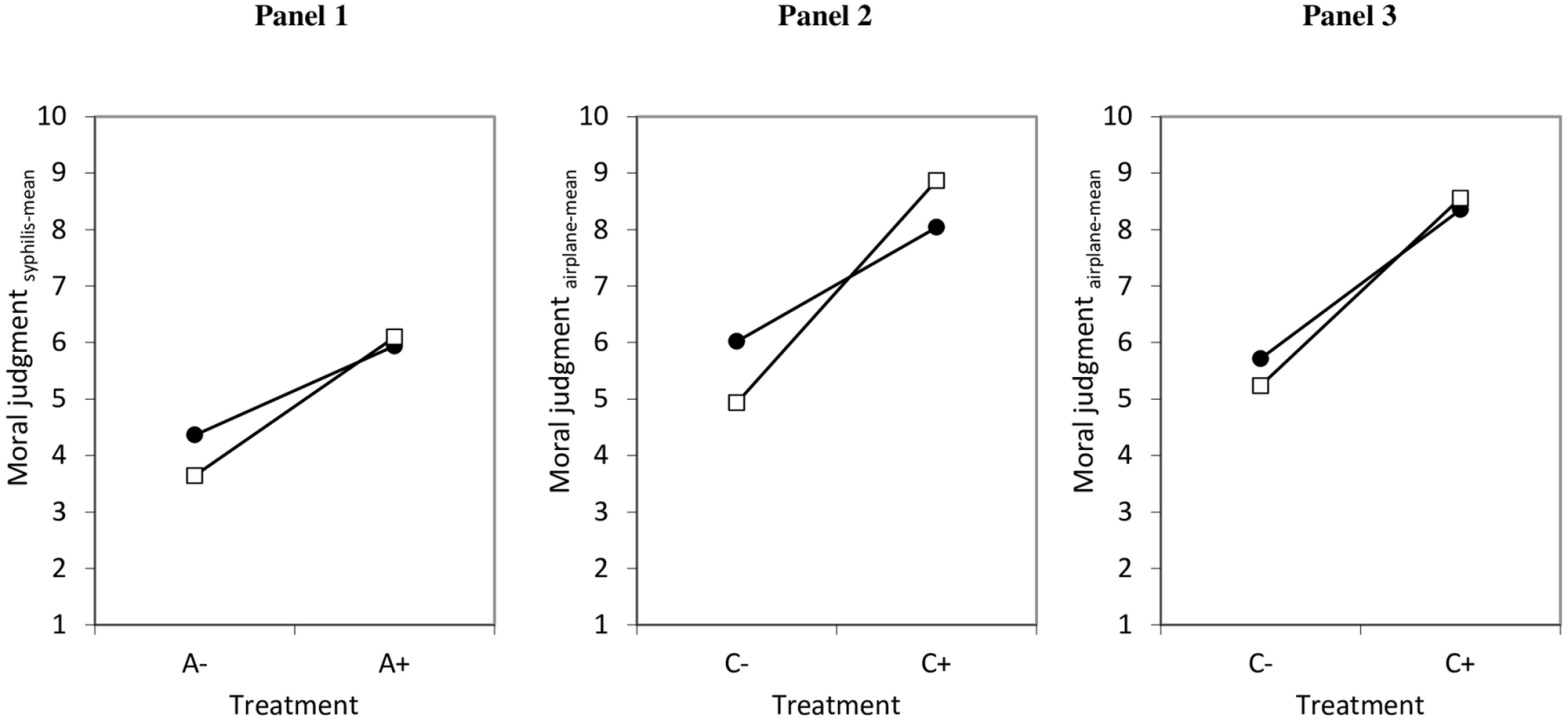
Predicted values for moral judgments depending on the experimental treatments and Preferences for Precepts Implied in Moral Theories (PPIMT) based on linear regression models.

In M_2_ and M_4_, we tested whether people with different PPIMT reacted differently to the variations of the ADC-components. M_2_ reveals a significant positive interaction effect between A and PPIMT_Virtue ethics_ for the low-stakes vignette (*p*_*SYPHILIS*_ = 0.017). This effect is shown in Panel 1 in Fig 5. If the PPIMT_Virtue ethics_ are high, A has a positive effect on moral judgement which is stronger than if these preferences are low (line with cuboids is steeper than line with dots). It seems that a negative description of the agent (A-) is judged more negative when the PPIMT_Virtue ethics_ are high, while a positive description of the agent (A+) is only minimally affected by PPIMT_Virtue ethics_.

M_4_, for the high-stakes vignette, shows a positive interaction between PPIMT_Deontology_ and C (*p*_*AIRPLANE*_ = 0.033). If the PPIMT_Deontology_ are high, C has a positive effect on moral judgement which is stronger than if these preferences are low (line with cuboids is steeper than line with dots, see Panel 2). It can be seen that the acceptability of a negative description of consequences (C-) is judged more negative if the PPIMT_Deontology_ are high rather than low, while a positive description of consequences (C+) is judged more positively if the PPIMT_Deontology_ are high rather than low.

M_4_ also shows a significant positive interaction effect between PPIMT_Consequentialism_ and C (*p*_*AIRPLANE*_<0.001). If the PPIMT_Consequentialism_ are high, C has a positive effect on moral judgement which is stronger than if these preferences are low (line with cuboids is steeper than line with dots, see Panel 3). If the PPIMT_Consequentialism_ are high rather than low, a negative description of consequences (C-) is seen as slightly more negative, while a positive description of consequences (C+) is seen as slightly more positive.

### Discussion of experiment 2

*Replication of the effects from experiment 1 in a between-subject design and in an additional high-stakes situation*: The results of this experiment, which used a between-subject design, showed that the most important predictions of the ADC-model hold for the investigated low-stakes and high-stakes situations. It found that with positive descriptions of the A-, D-, and C-components, the situation is judged as being more acceptable. We replicated the findings of the differing strengths of the ADC-components in the low-stakes vignette: the C-component resulted in the smallest effect, followed by the A-component, with the D-component having the strongest effects. These results exclude the possibility that contrast and learning effects influenced the results of experiment 1. For the high-stakes vignette, the strongest effect was observed for the C-component (i.e., the pilot and the passengers die vs. they are saved), followed by D-component (i.e., crippling the hijacker vs. calming him), with the A-component (i.e., greedy vs. benevolent) having the smallest effect. These results exclude an a priori difference in intuitive strength of different components of moral judgment, and seem to indicate that they are context-sensitive. In addition to a replication of the main effects of A, D, and C, we were also able to generally replicate their interaction pattern for the low-stakes vignette (see interpretation above).

In the high-stakes scenario, we did not find the significant conditional positive two-way interaction effects between A and C found in the low-stakes scenario, what’s more, no interaction exists at all. Also the positive interaction effect between A and D that has been found before did not exist in the high-stakes scenario—both components interact negatively, but only if C is negative. Moreover, the conditional negative interaction effect between C and D could not be exactly replicated—in the high-stakes vignette such a negative interaction effect occurs if A is negative as compared to if A is positive in the low-stakes vignette. This might mean either that the level of stakes can affect the interplay between the ADC-components (e.g., respondents might partially resist the intuitive pull in line with cautionary proverbs, such as ‘the road to hell is paved with good intentions’), or that the situation of the vignettes themselves influenced the interaction effects (see e.g., [23]: 928). Indeed, Brady & Wheeler ([23]:934) report that biasing towards consequentialist or deontological responses is “situation-specific”, i.e., dependent on the description of individual vignettes used in research. Ultimately, more research with different situations with different stakes should complement or challenge these first results of the ADC-model in which all components have been operationalized simultaneously. Nevertheless, our results indicate the great interest and value of pursuing research on a three-component model of moral judgment in comparison to simpler models.

*Effects of PPIMT*: Prior research has found that ethical predispositions (a construct that is conceptually similar to our PPIMT) can affect the perception of whether a situation contains moral content [24]. We found hardly any main effects for respondents with different preferences. We only found that people with stronger PPIMT_Virtue ethics_ judge the moral acceptability of the high-stakes scenario as being higher—irrespective of the variations of A, D, and C. We have no general interpretation for this effect and the lack of main effects of PPIMT_Deontology_ and PPIMT_Consequentialism_. But results indicate that situational characteristics seem to matter more than respondent characteristics alone.

However, we found three interaction effects between the PPIMT and the characteristics of the situation, namely variations of the A- and C-components. We found two such interaction effects between “intra-theoretic” variables (i.e., effects within the purview of one moral theory). In the low-stakes scenario, the effect of A was stronger if respondents had higher PPIMT_Virtue ethics_. This seems to be due to the observation that a negative description of the agent (i.e., who is in opposition to this preference) is judged less acceptable among respondents with high rather than low PPIMT_Virtue ethics_. One explanation could be that respondents with such preferences might actively look for corresponding cues and if these cues violate their preference, the situation is judged as less acceptable. Another, not mutually exclusive, explanation is that these respondents are influenced by such cues since the situation activates their preferences and then guides their more negative judgments.

In the high-stakes scenario, the effect of C was higher for respondents with high rather than low PPIMT_Consequentialism_: if the consequences match the preferences, i.e., better consequences are judged more positively; if the consequences do not match the preferences, i.e., negative consequences are judged more negatively. This means that a respondent’s PPIMT_Consequentialism_ strengthens the effects of the variations of the situations in both directions. The interpretation of this is similar to the one for the interaction effect reported before. No more interaction effects between “intra-theoretic” variables were found in any of the other vignette variants. Thus, caution should be exercised when interpreting these results, especially since these two effects were not observed 1) in both vignettes; 2) for other preferences for precepts implied in a certain moral theory and components of the model; and 3) in both directions.

Apart from these two interaction effects between “intra-theoretic” variables, we also observed one interaction effect between “theoretic” variables (i.e., an effect across moral theories). In the high-stakes scenario, respondents with high PPIMT_Deontology_ reacted stronger to variations in the C component: i.e., better consequences are judged more positively; while negative consequences are judged more negatively. Deontology as a moral theory specifically dictates that acts be judged based on principles of duty and obligation, whatever the consequences may be. In practice, this leads to many counter-intuitive conclusions, such as a moral prohibition on lying, even if it would save numerous lives. However, apart from professional moral philosophers who defend single-component deontological theories, few people are genuinely inclined to disregard consequences entirely (see [21]), even if they have PPIMT_Deontology_. Our findings are in line with some of the predictions of the ADC model. Namely, unlike single-component moral theories, the ADC model postulates that sound moral judgment needs to take into account all three sources of moral knowledge. Thus, people socialized to prefer deontological precepts are intuitively highly influenced by the assessment of consequences—the very aspect they are supposed to ignore. It may be the case that the imperative ‘to ignore consequences’ is precisely what makes the C component more salient. All in all, our results invite more caution in research about ethical ‘frameworks’ given the complexity that we discovered.

*Lack of socio-demographics effects*: As in experiment 1, we found no significant effects of sex, age, education, and subjective social status. This result might seem surprising given the longstanding claims that moral judgments are influenced by a person’s gender (see [22]) or age, in terms of stages of development (see [47]). However, only mixed results for the impact of gender and age on moral judgment have been found in several comprehensive literature reviews [46]. For instance, gender has been investigated in many studies in recent years [46,48,49,50] but results imply that no clear effects of gender show a continual trend in various populations and cultural groups [46]. However, more research would be necessary before firm conclusions on the irrelevance of all our investigated socio-demographics can be made. For instance, it is possible that age and gender play a more important role in earlier stages of moral development and that our sample (comprised of individuals aged 20 to 77), similarly to other studies in adult population, simply could not reflect effects that are significant at earlier ages (e.g., 12–19). Future work should verify whether results of our subjective measure of SES can be replicated with more objective measures (such as income and occupation). However, we found that one common objective measure, namely, education did not reveal different results than the subjective measure. Finally, although the sample size of the present study is adequate, all participants came from a single Western society, and this limits the generalizability about the lack of effects of socio-demographic variables. Future research with transcultural samples needs to be conducted.

## General discussion

There is a growing sense that moral intuitions represent the building blocks of moral judgment, but competing theories about the relationship between moral intuitions and moral judgment continue to be debated (see e.g., [2]). The ADC-model of moral judgment posits that moral judgment relies on three components of morality (agent-; deed-, and consequence-based evaluations) [9,10]. It relates mechanisms for moral intuition to established moral theories, as well as general theories of intuitive decision making. This model explains the considerable flexibility and stability of moral judgment. However, insistence on single-component explanations has led to many centuries of debate as to which moral precepts and theories best describe (or should guide) moral evaluation. The ADC-model provides an account in which the use of all three components is a hallmark of sound moral judgment. In immature and under-developed individuals, moral evaluation is rigid [16,28,51], whereas in certain populations (most notably with frontal lobe pathology) it seems to be guided by only a subset of morally relevant aspects, notably consequences [52–54], which in turn leads to aberrant judgment and behavior.

As predicted by the ADC-model, in experiment 1 (based on a within-subject design), we showed that each component of moral intuition significantly contributes to moral judgment in a low-stakes vignette. The between-subject design of experiment 2 yielded results confirming and replicating the main effects of A, D, and C, as well as their patterns of interaction effects for the low-stakes vignette. Experiment 2 also suggests that A, D, and C play a significant role in high-stakes vignettes. However, the relative strength of the effects of the components differed across vignettes: in the low-stakes vignette, D had the strongest effect, followed by A, and then C; in the high-stakes vignette, C had the strongest effect, followed by D, and then A. Moreover, the pattern of interaction effects also differed in the high-stakes vignette.

In experiment 2, we also found that PPIMT_Virtue ethics_, PPIMT_Deontology_, and PPIMT_Consequentialism_ selectively moderated the effects of the characteristics of the situation. However, these effects are not as robustly observed for all PPIMT as are the main effects of A, D, and C and this further supports the status of A, D, and C as fundamental components of moral judgment. In addition, in both experiments we found no significant effects of the investigated socio-demographics (i.e., sex, age, education, and subjective social status) on moral judgment. This first experimental investigation of the ADC-model offers insights into intriguing issues with respect to normative ethics and its relationships to moral psychology and cognitive science, which we discuss below.

### Relationship between moral intuition and moral judgment

The content of moral intuition and its connection to normative ethics is hotly debated (see [6,8,9]). The present evidence supports the novel explanation proposed by the ADC model of moral judgment for the intuitive evaluations: different components of moral intuitions (A, D, and C) drive moral judgment. For instance, if respondents are asked “Is it OK to lie?” or “Is it OK to commit incest?”, these questions are specifically (and mainly) probing the D component, and not the overall intuitive judgment, which is comprised of the three ADC-components. However, our results show the interest and value of a three-component solution. Accordingly, future studies into moral intuitions and moral judgment may build from this fundamental insight.

### Flexibility and stability of moral intuitions

Much of the research in experimental moral psychology has been motivated by the desire to prove one normative moral theory correct (e.g., consequentialism) and undermine the others (see [20,55]) with the hope of explaining the stability of moral judgment. However, such explanations have always run into difficulties in explaining the flexibility of moral judgments and the integration of different components of moral intuition. The experiments we report suggest an explanation for the perplexing flexibility and stability of moral intuitions. Contrary to traditional disputes about single-component theories in moral philosophy, and to some extent in moral psychology, our results suggest that intuitions underlying precepts from all three dominant moral theories (virtue ethics, deontology, and consequentialism) are operative (in terms of a stable and significant contribution of each component of moral judgment). However, none of the components are overriding in the sense that a single component is making the contribution of both other components irrelevant. Furthermore, the results also show that there are instances in which the combination of two components can render the influence of the third component less relevant as predicted by the ADC model. Interestingly, the D-component, was never rendered less relevant by the other components, thus, the kind of deed one performs seems to be of fundamental relevance for moral judgement. These results are important in moving forward.

### Effect of PPIMT

A recent trend in empirical moral psychology has emphasized the potential ineffectiveness of moral reasoning [3] or knowledge about ethics [56], while there is a significant body of literature on the effects of predispositions toward certain moral theories or ethical frameworks (see, e.g., [21,23,24,46]). The experiments reported here provide evidence against the assumption that PPIMT have no effect on moral judgment. We found that preferences for certain precepts implied in the three dominant moral theories exert selective differential interaction effects with the A- and C-components. Namely, if a person believes that virtues matter, we found evidence in the low-stakes vignette that, in situations in which an agent is described as non-virtuous and thus falls short of the virtuous expectations, stronger PPIMT_Virtue ethics_ lead to a more negative evaluation of the situation. A possible alternative interpretation is that the characteristics of the situation (i.e., the variation of A) activates a person’s preferences and guides them to make moral judgments in accordance with their preferences. Similarly, the stronger a PPIMT_Consequentialism_, the more weight respondents gave to positive and negative consequences. Finally, those who have PPIMT_Deontology_ might be primed by the deontological imperative to ‘ignore consequences’, which makes the C component more salient. More research is needed to test these interpretations and our newly developed PPIMT instrument to measure preferences for the precepts implied in a certain moral theory (see below) can be a means to tease out which processes are at play at the level of such preferences.

### Methodological improvements

Finally, another contribution of this study is that it offers an additional way for investigating three components of intuitive moral judgment simultaneously instead of one or two, processes for developing validated vignettes, as well as a new instrument to examine the preferences entailed by three distinct moral theories (i.e., the PPIMT). Currently, most data obtained from current moral psychology research focuses on the use of so-called “trolley dilemmas”, highly unrealistic moral scenarios involving runaway trolleys. In contrast, the scenarios and methods developed in this study and the ADC-model aim to contribute to more valid investigation of moral evaluation and moral judgment. For example, only two of the seven scenarios initially developed successfully passed the multiple stages of pretesting we undertook. This is a methodological strength but it implies a limited resulting number of validated scenarios, a limitation which future studies will address. Furthermore, by going beyond the operationalization of one preferred moral theory or pitting predictions from two moral theories against each other, our study provides an alternative tool to test predictions from the three dominant moral theories simultaneously. We developed the PPIMT-instrument with good statistical properties, which allows for differential assessment of preferences for the moral precepts implied in virtue ethics, deontology, and consequentialism—and thus could be used to clarify ‘mixed’ preferences (e.g., preference for utilitarian solutions and deontological rationales, or vice versa) reported in the ethical decision-making literature. A potential limitation of the PPIMT-instrument is that similar wording of items falling under a given theory could have contributed to the clustering of items. Despite this limitation, the scale is a ‘proof of concept’–showing that three moral theories can be operationalized simultaneously, and future research should focus on developing further items that address this potential limitation. We hope that these methodological improvements we have offered will further the investigation of moral judgment.

## Conclusion

There is increasing consensus in moral psychology and theory that most moral judgments are guided by unconscious intuitive evaluations. However, questions about the processes driving intuitive appraisals, as well as the role of moral theories and their precepts, have been answered in different ways [57]. The ADC-model explains the emergence of moral judgments by the processing of three components (evaluations of Agents, Deeds, and Consequences). This first empirical investigation of the ADC-model suggests that these components that guide moral evaluations are consistently employed, and that precepts implied in virtue ethics, deontology and consequentialism are closely aligned with these sources of moral knowledge. Overall, our results offer a strong empirical corroboration of the ADC-model of moral judgment [9,10], which provides an explanation for the intuitive appeal of dominant moral theories. Finally, our study provides support for the long-held belief that intuitive moral judgment is a good starting point for grounding philosophical inquiry and moral reasoning, even though it has frustrated efforts at successfully grounding intuitive appeal of moral theories by ignoring other components (e.g., utilitarianism).

## Supporting information

S1 Text Overview and critique of the Universal Moral Grammar model. (DOCX)

S2 Text Overview and critique of the Moral Foundations Theory. (DOCX)

S1 Table T-tests for the manipulation checks for low- and high-stakes vignettes. (DOCX)

S2 Table Correlation coefficients between self-identifications with moral theories and Preferences for Precepts Implied in Moral Theories (PPIMT). (DOCX)

S3 Table Summary of the findings of the ADC-components. (DOCX)

S1 File Pretest dataset. (DTA)

S2 File Experiment 1 dataset. (DTA)

S3 File Experiment 2 dataset. (DTA)
