# Correction: Deciphering moral intuition: How agents, deeds, and consequences influence moral judgment

**DOI:** 10.1371/journal.pone.0206750

**Published:** 2018-10-25

**Authors:** Veljko Dubljević, Sebastian Sattler, Eric Racine

The Supporting Information files S1 and S2 Text and S1, S2 and S3 Tables incorrectly contain tracked changes. Please view the corrected files below.

## Supporting information

S1 Text Overview and critique of the Universal Moral Grammar model. (DOCX)

S2 Text Overview and critique of the Moral Foundations Theory. (DOCX)

S1 Table T-tests for the manipulation checks for low- and high-stakes vignettes. (DOCX)

S2 Table Correlation coefficients between self-identifications with moral theories and Preferences for Precepts Implied in Moral Theories (PPIMT). (DOCX)

S3 Table Summary of the findings of the ADC-components. (DOCX)
