# Supplementary material for: Deciphering moral intuition: How agents, deeds, and consequences influence moral judgment
Source: PLoS One. 2018 Oct 1;13(10):e0204631. doi: 10.1371/journal.pone.0204631 (PMC6166963; doi:10.1371/journal.pone.0204631)
Supplement: S1 Text — (DOCX) [file pone.0204631.s001.docx]

**S1 Text. Overview and critique of the Universal Moral Grammar model**

The Universal Moral Grammar model explains moral intuition as unconscious logical structures which scaffold moral judgment according to certain rules focusing on avoiding intentional harm, as specified by the principle of double effect. This principle offers a mid-level moral rule as an explanation for inconsistencies between general precepts of consequentialism, virtue ethics, and deontology, and specifies that it is worse to harm a person as a *means* to saving others than to harm a person as a *side effect* of saving others [56]. Universal Moral Grammar seeks to describe the nature and origin of moral knowledge by using concepts and models similar to those used in the study of language: according to this model, intuitive morality relies on a complex set of operations, including (i) identifying the various action descriptions in the stimulus, (ii) placing them in an appropriate temporal order, (iii) decomposing them into their underlying causative and semantic structures, (iv) applying certain moral and logical principles to these underlying structures to generate representations of good and bad effects, (v) computing the intentional structure of the relevant acts and omissions by inferring (in the absence of conflicting evidence) that agents intend good effects and avoid bad ones, and (vi) deriving representations of morally salient acts like battery and situating them in the correct location of one's act tree [7]. Thus, according to Universal Moral Grammar, a single moral judgment ultimately depends on whether harm was intended or not: in cases where lying is not accompanied with intended good effects (such as avoidance of harm), it is considered morally unacceptable, whereas if it is a condition for saving lives, it is morally acceptable. Again, there is some evidence to support this model [8], but there is no direct way to test if these complex operations are actually occurring as (implicit or explicit) mental representations during moral judgment tasks. Most notably, the complexity of experimentally examining the proposed cascade of cognitive processes prevents the gathering of conclusive evidence at this time. Ultimately, Universal Moral Grammar suggests the existence of improbable mechanisms specific to moral cognition, separate from other cognitive processes and heuristics, rather than understanding moral intuitions in light of what is known about intuitive decision making more broadly.
