# Supplementary material for: Deciphering moral intuition: How agents, deeds, and consequences influence moral judgment
Source: PLoS One. 2018 Oct 1;13(10):e0204631. doi: 10.1371/journal.pone.0204631 (PMC6166963; doi:10.1371/journal.pone.0204631)
Supplement: S2 Text — (DOCX) [file pone.0204631.s002.docx]

**S2 Text. Overview and critique of the Moral Foundations Theory**

According to the Moral Foundations Theory (MFT), moral intuition is emotionally modulated and cued by specific innate, basic moral domains: Harm/Care, Fairness, Loyalty, Authority, and Purity [6]. Each moral domain ostensibly has prototypical positive and negative prompts, which in turn trigger specific responses, which are the bases of culturally variable virtues. The domain of harm is the most basic drive to avoid hurting others according to which moral intuitions respond to the failure to care, and also duties of cherishing and protecting others. The domain of fairness allegedly involves rendering justice according to shared rules, and moral intuitions in this domain mostly respond to instances of perceived cheating. The domain of loyalty supposedly entails standing with one’s group, family, or nation, and moral intuitions in this domain mostly respond to instances of perceived betrayal. The domain of authority purportedly implies submitting to tradition and legitimate leadership and moral intuitions in this domain mostly respond to instances of perceived subversion or disrespect. Finally, the purity domain ostensibly involves abhorrence for disgusting things, foods, actions and people, and moral intuitions in this domain mostly respond to instances of perceived degradation. So, according to MFT, a single moral judgment ultimately depends on which moral domain is the most salient in any given situation. As in the example of lying from the introduction, when lying is not accompanied by other moral issues (such as harm), the fairness intuition evaluates deceit as morally unacceptable, whereas if lying saves lives, the intuition from the domain of care/harm takes precedence, resulting in an evaluation of deceit as morally acceptable. However, testing these explanations in different lines of empirical inquiry has not yielded definitive support for the hypotheses and the evidence is far from being unequivocal or conclusive, most notably because the strong role of emotion in moral judgments has not been shown to follow a simple causal trajectory as was predicted by MFT (see e.g., [57]). Furthermore, many of the purported foundational moral domains reflect influences on moral judgment which are inconsistent with core insights of several of the major moral theories. Indeed, MFT appears to confuse moral intuitions with social biases to the point where they seem almost completely unreliable as a means to guide or inform more complex moral cognitive processes, such as philosophical reflection done by experts or, for example, jury deliberation done by non-experts in criminal proceedings.
