# Supplementary material for: Deciphering moral intuition: How agents, deeds, and consequences influence moral judgment
Source: PLoS One. 2018 Oct 1;13(10):e0204631. doi: 10.1371/journal.pone.0204631 (PMC6166963; doi:10.1371/journal.pone.0204631)
Supplement: S1 Table — (DOCX) [file pone.0204631.s003.docx]

**S1 Table. T-tests for the manipulation checks for low- and high-stakes vignettes.**

|  | ***Total N*** |  | **Negative (-)** | | | **Positive (+)** | | |  | ***t-value*** |
| --- | --- | --- | --- | --- | --- | --- | --- | --- | --- | --- |
|  |  |  | ***N*** | ***M*** | ***SD*** | ***N*** | ***M*** | ***SD*** |  |  |
| ***Low-stakes (Syphilis)*** |  |  |  |  |  |  |  |  |  |  |
| A | 47 |  | 17 | 0.72 | 0.624 | 30 | -0.41 | 1.003 |  | 4.37^***^ |
| D | 51 |  | 25 | 0.73 | 0.748 | 26 | -0.70 | 0.654 |  | 7.25^***^ |
| C | 58 |  | 29 | 0.29 | 1.162 | 29 | -0.26 | 0.739 |  | 2.02^***^ |
| ***High-stakes (Airplane)*** |  |  |  |  |  |  |  |  |  |  |
| A | 46 |  | 24 | 0.35 | 1.204 | 22 | -0.39 | 0.508 |  | 2.67^***^ |
| D | 52 |  | 32 | 0.45 | 1.023 | 20 | -0.71 | 0.299 |  | 4.91^***^ |
| C | 59 |  | 28 | 0.55 | 0.749 | 31 | -0.47 | 1.002 |  | 4.50^***^ |

*Notes:* ^*^ *p*<0.05, ^**^ *p*<.01, ^***^ *p*<.001. *N*=Number of observations, *M*=Mean value; *SD*=Standard deviation.
