# Supplementary material for: Deciphering moral intuition: How agents, deeds, and consequences influence moral judgment
Source: PLoS One. 2018 Oct 1;13(10):e0204631. doi: 10.1371/journal.pone.0204631 (PMC6166963; doi:10.1371/journal.pone.0204631)
Supplement: S2 Table — (DOCX) [file pone.0204631.s004.docx]

**S2 Table. Correlation coefficients between self-identifications with moral theories and Preferences for Precepts Implied in Moral Theories (PPIMT).**

|  | 1 | 2 | 3 | 4 | 5 |
| --- | --- | --- | --- | --- | --- |
| 1: Self-identification_Virtue ethicist_ | ─ |  |  |  |  |
| 2: Self-identification_Deontologist_ | 0.161  (146) | ─ |  |  |  |
| 3: Self-identification_Consequentialist_ | -0.035  (146) | -0.083  (147) | ─ |  |  |
| 4: PPIMT_Virtue ethics_ | 0.395***  (135) | 0.122  (138) | -0.147  (138) | ─ |  |
| 5: PPIMT_Deontology_ | -0.106  (135) | 0.476***  (138) | -0.073  (138) | 0.000  (141) | ─ |
| 6: PPIMT_Consequentialism_ | -0.035  (135) | -0.107  (138) | 0.480***  (138) | 0.000  (141) | 0.000  (141) |

*Note: * p*<.05. ** *p*<.01. *** *p*<.001; Number of observations in brackets.
