# Supplementary material for: Deciphering moral intuition: How agents, deeds, and consequences influence moral judgment
Source: PLoS One. 2018 Oct 1;13(10):e0204631. doi: 10.1371/journal.pone.0204631 (PMC6166963; doi:10.1371/journal.pone.0204631)
Supplement: S3 Table — (DOCX) [file pone.0204631.s005.docx]

**S3 Table. Summary of the findings of the ADC-components.**

| **Experiment 1: Syphilis** | **Experiment 2: Syphilis** | **Experiment 2: Airplane** |
| --- | --- | --- |
| Effects of A | Effects of A | Effects of A |
| *Strength: Medium effect* | *Strength: Medium effect* | *Strength: Smallest effect* |
| *Simple slope tests* | *Simple slope tests* | *Simple slope tests* |
| line 1 D+C+): sig. positive | line 1 D+C+): sig. positive | line 1 D+C+): no effect |
| line 2 (D+C-): sig. positive | line 2 (D+C-): sig. positive | line 2 (D+C-): sig. positive |
| line 3 (D-C+): sig. positive | line 3 (D-C+): sig. positive | line 3 (D-C+): sig. positive |
| line 4 (C-D-): sig. positive | line 4 (C-D-): no effect | line 4 (C-D-): sig. positive |
|  |  |  |
| *Slope difference tests* | *Slope difference tests* | *Slope difference tests* |
| line 1 (D+C+) = line 2 (D+C-) | line 1 (D+C+) = line 2 (D+C-) | line 1 (D+C+) = line 2 (D+C-) |
| line 1 (D+C+) > line 3 (D-C+) | line 1 (D+C+) > line 3 (D-C+) | line 1 (D+C+) = line 3 (D-C+) |
| line 1 (D+C+) > line 4 (C-D-) | line 1 (D+C+) > line 4 (C-D-) | line 1 (D+C+) = line 4 (C-D-) |
| line 2 (D+C-) > line 3 (D-C+) | line 2 (D+C-) > line 3 (D-C+) | line 2 (D+C-) = line 3 (D-C+) |
| line 2 (D+C-) > line 4 (C-D-) | line 2 (D+C-) > line 4 (C-D-) | line 2 (D+C-) < line 4 (C-D-) |
| line 3 (D-C+) > line 4 (C-D-) | line 3 (D-C+) > line 4 (C-D-) | line 3 (D-C+) = line 4 (C-D-) |
|  |  |  |
| Effects of D | Effects of D | Effects of D |
| *Strength: Largest effect* | *Strength: Largest effect* | *Strength: Medium effect* |
|  |  |  |
| *Simple slope tests* | *Simple slope tests* | *Simple slope tests* |
| line 1 (A+C+): sig. positive | line 1 (A+C+): sig. positive | line 1 (A+C+): sig. positive |
| line 2 (A-C+): sig. positive | line 2 (A-C+): sig. positive | line 2 (A-C+): sig. positive |
| line 3 (A+C-): sig. positive | line 3 (A+C-): sig. positive | line 3 (A+C-): sig. positive |
| line 4 (A-C-): sig. positive | line 4 (A-C-): sig. positive | line 4 (A-C-): sig. positive |
|  |  |  |
| *Slope difference tests* | *Slope difference tests* | *Slope difference tests* |
| line 1 (A+C+) > line 2 (A-C+) | line 1 (A+C+) > line 2 (A-C+) | line 1 (A+C+) = line 2 (A-C+) |
| line 1 (A+C+) < line 3 (A+C-) | line 1 (A+C+) < line 3 (A+C-) | line 1 (A+C+) = line 3 (A+C-) |
| line 1 (A+C+) > line 4 (A-C-) | line 1 (A+C+) > line 4 (A-C-) | line 1 (A+C+) < line 4 (A-C-) |
| line 2 (A-C+) < line 3 (A+C-) | line 2 (A-C+) < line 3 (A+C-) | line 2 (A-C+) = line 3 (A+C-) |
| line 2 (A-C+) = line 4 (A-C-) | line 2 (A-C+) = line 4 (A-C-) | line 2 (A-C+) < line 4 (A-C-) |
| line 3 (A+C-) > line 4 (A-C-) | line 3 (A+C-) > line 4 (A-C-) | line 3 (A+C-) = line 4 (A-C-) |
|  |  |  |
| Effects of C | Effects of C | Effects of C |
| *Strength: Smallest effect* | *Strength: Smallest effect* | *Strength: Largest effect* |
|  |  |  |
| *Simple slope tests* | *Simple slope tests* | *Simple slope tests* |
| line 1 (A+D+): sig. positive | line 1 (A+D+): marginal sig. | line 1 (A+D+): sig. positive |
| line 2 (A-D+): no effect | line 2 (A-D+): sig. positive | line 2 (A-D+): sig. positive |
| line 3 (A+D-): sig. positive | line 3 (A+D-): sig. positive | line 3 (A+D-): sig. positive |
| line 4 (A-D-): sig. positive | line 4 (A-D-): sig. positive | line 4 (A-D-): sig. positive |
|  |  |  |
| *Slope difference tests* | *Slope difference tests* | *Slope difference tests* |
| line 1 (A+D+) = line 2 (A-D+) | line 1 (A+D+) = line 2 (A-D+) | line 1 (A+D+) = line 2 (A-D+) |
| line 1 (A+D+) < line 3 (A+D-) | line 1 (A+D+) < line 3 (A+D-) | line 1 (A+D+) = line 3 (A+D-) |
| line 1 (A+D+) = line 4 (A-D-) | line 1 (A+D+) = line 4 (A-D-) | line 1 (A+D+) = line 4 (A-D-) |
| line 2 (A-D+) < line 3 (A+D-) | line 2 (A-D+) < line 3 (A+D-) | line 2 (A-D+) < line 3 (A+D-) |
| line 2 (A-D+) = line 4 (A-D-) | line 2 (A-D+) = line 4 (A-D-) | line 2 (A-D+) < line 4 (A-D-) |
| line 3 (A+D-) > line 4 (A-D-) | line 3 (A+D-) > line 4 (A-D-) | line 3 (A+D-) = line 4 (A-D-) |
|  |  |  |

*Note: “*<” the ascent of the line before the sign is steeper than the line behind (respectively “=” equal or flatter “>”).
